# Genetic variants of programmed cell death 1 are associated with HBV infection and liver disease progression

**DOI:** 10.1038/s41598-021-87537-9

**Published:** 2021-04-08

**Authors:** Nghiem Xuan Hoan, Pham Thi Minh Huyen, Mai Thanh Binh, Ngo Tat Trung, Dao Phuong Giang, Bui Thuy Linh, Dang Thi Ngoc Dung, Srinivas Reddy Pallerla, Peter G. Kremsner, Thirumalaisamy P. Velavan, Mai Hong Bang, Le Huu Song

**Affiliations:** 1 Department of Blood-Borne Infectious Diseases, Institute of Clinical Infectious Diseases, 108 Institute of Clinical Medical and Pharmaceutical Sciences, Hanoi, Vietnam; 2 Vietnamese-German Center for Medical Research (VG-CARE), Hanoi, Vietnam; 3 Department of Biochemistry, 108 Institute of Clinical Medical and Pharmaceutical Sciences, Hanoi, Vietnam; 4 Faculty of Gastroenterology, 108 Institute of Clinical Medical and Pharmaceutical Sciences, Hanoi, Vietnam; 5 Centre for Genetic Consultation and Cancer Screening, 108 Institute of Clinical Medical and Pharmaceutical Sciences, Hanoi, Vietnam; 6 Hanoi Medical University, Hanoi, Vietnam; 7 Institute for Tropical Medicine, University Hospital Tübingen, Tübingen, Germany; 8 Centre de Recherches Médicales de Lambaréné (CERMEL), Lambaréné, Gabon

**Keywords:** Molecular medicine, Clinical genetics, Genotype, Haplotypes, Medical genetics, Infectious diseases

## Abstract

The inhibitory effects of programmed cell death 1/programmed cell death ligand 1 (PD-1/PD-L1) modulates T-cell depletion. T-cell depletion is one of the key mechanisms of hepatitis B virus (HBV) persistence, in particular liver disease progression and the development of hepatocellular carcinoma (HCC). This case–control study aimed to understand the significance of PD-1 polymorphisms (*PD-1.5 and PD-1.9*) association with HBV infection risk and HBV-induced liver disease progression. Genotyping of *PD-1.5* and *PD-1.9* variants was performed by direct Sanger sequencing in 682 HBV-infected patients including chronic hepatitis (CHB, n = 193), liver cirrhosis (LC, n = 183), hepatocellular carcinoma (HCC, n = 306) and 283 healthy controls (HC). To analyze the association of *PD-1* variants with liver disease progression, a binary logistic regression, adjusted for age and gender, was performed using different genetic models. The *PD-1.9 T* allele and *PD-1.9 TT* genotype are significantly associated with increased risk of LC, HCC, and LC + HCC. The frequencies of *PD-1.5 TT* genotype and *PD-1.5 T* allele are significantly higher in HCC compared to LC patients. The haplotype *CT* (*PD-1.5 C* and *PD-1.9 T*) was significantly associated with increased risk of LC, HCC, and LC + HCC. In addition, the *TC* (*PD-1.5 T* and *PD-1.9 C*) haplotype was associated with the risk of HCC compared to non-HCC. The *PD-1.5 CC, PD-1.9 TT,* genotype, and the *CC* (*PD-1.5 C* and *PD-1.9)* haplotype are associated with unfavorable laboratory parameters in chronic hepatitis B patients. *PD-1.5* and *PD1.9* are useful prognostic predictors for HBV infection risk and liver disease progression.

## Introduction

Chronic hepatitis B virus (CHB) infection causes long-term clinical complications, including acute liver failure, liver cirrhosis (LC) and hepatocellular carcinoma (HCC). WHO estimates 257 million cases of CHB and 780,000 HBV-related deaths annually^1^. Although an effective vaccination program has reduced the burden of hepatitis B virus (HBV), the persistence of CHB in adult populations remains a significant health burden, particularly in low and middle-income countries, including Vietnam^1,2^. The incidence of HBV is > 10% in the Vietnamese population and is one of the major contributors of mortality^1^.

During the clinical course of HBV, both innate and adaptive immune responses largely modulate the liver disease progression and subsequent liver damage. Several compelling evidences reveal that human cellular immune response is crucial for HBV pathogenesis and such responses are mediated by CD8^+^ and CD4^+^T cells, especially by HBV-specific CD8^+^T cells^3–7^. HBV clearance is arbitrated by HBV-specific CD8^+^T cells, by cytopathic and noncytolytic activity^7–9^, largely during the acute phase. A relatively weak HBV-specific CD8^+^T cell response was equally observed during the chronic phase^4,10–13^. The HBV-specific CD8^+^T cell depletion occurs primarily by the activation of the inhibitory checkpoint PD-1/PD-L1 (Programmed cell death 1/Programmed cell death ligand 1) signaling pathway^14–20^.

PD-1 is a transmembrane protein and an immunoinhibitory receptor, that are largely expressed on the surface of T cells, B cells, natural killer T cells and activated monocytes^21–23^. The PD-1 immunomodulatory ligands (PD-L1 and PD-L2) maintain immune homeostasis, but can also attenuate T-cell proliferation and regulate cytokine responses^24^. The phenomenon of persistent overexpression of PD-1 and its ligands is a common feature in chronic infections and cancer^25–27^, and blocking inhibitory signal transduction has led to increased T-cell function, and thus improved clinical outcomes. Blocking the PD-1/PD-L1 signaling with anti PD-1 or anti-PD-L1 molecules in combination with antiviral therapies has led to better immunological and clinical responses in HBV infections^28,29^. Clinical studies have also used expression of PD-1 and CTLA-4 as prognostic indicators, in evaluating antiviral HBV therapy responses^30^.

Several genetic association studies had investigated genetic variants of programmed cell death 1 (*PD-1*) and programmed death ligand 1 (*PD-L1*) with cancer, autoimmune and infectious diseases in different world populations^31–35^. In particular*,* five single nucleotide polymorphisms of *PD1: PD-1.1* (-538G/A*), PD-1.3* (+ 7146G/A), *PD-1.5* (+ 7785T/C), *PD-1.6* (+ 8669G/A) *and PD-1.9* (+ 7625C/T) were associated with human malignancies and is highly expressed in several cancers^32,36–39^. Genetic variations in the promoter and at the transcription binding site can alter gene function. The *PD-1.1* (-538G/A*)* promoter variant, located on the translation start codon, while *PD-1.3* (+ 7146A/G) is located on intron 4, has been described to alter the gene transcription, since there are four tandem repeats consisting of several putative binding sequences of transcription factors^40^. *PD-1.5* (+ 7785T/C), a synonymous mutation in exon 5, which is in linkage equilibrium with other *PD-1* variants^31^. Relatively few studies have investigated on the association of *PD-1* variants in CHB infection and liver disease progression, but the results are debatable^41–45^.

The investigated *PD-1* variants are located in the 5′ upstream regions of the PD-1 locus (Genbank ID: 5133). The variant *PD-1.5* (rs2227981) is associated with cancer susceptibility^46^; while the variant *PD-1.9 (*rs2227982*)* is associated with risk of HBV infection and clinical outcome^47^. In this context, we conducted a case–control study with the aim to determine the association of *PD1* (*PD-1.5 and PD-1.9)* variants with HBV infection risk, liver disease progression and clinical outcome in the Vietnamese population.

## Materials and methods

### Ethics statement

This study was performed in accordance with the relevant national guidelines and regulations. Informed written consent was obtained from all participants after explanation of the study at the time of sampling. Informed consent was obtained from a parent and/or legal guardian for minors. The study was approved by the institutional review board of the 108 Institute of Clinical Medical and Pharmaceutical Sciences, Hanoi, Vietnam.

### Patients and liver specimens

A total of 682 unrelated Vietnamese HBV-infected patients were randomly recruited in a case–control design at 108 Institute of Clinical Medical and Pharmaceutical Sciences, Hanoi, Vietnam, between 2012 and 2015. Patients were assigned to subgroups of disease based on clinical manifestations, liver function tests, imaging modalities and histological confirmation for all HCC cases. Subgroups were chronic hepatitis (CHB, n = 193), liver cirrhosis (LC, n = 183), hepatocellular carcinoma (HCC, n = 306). The diagnostic criteria for the CHB patients and the HBV-related LC were previously described^48^. The HBV-related HCC group was characterized as patients infected with HBV and was diagnosed based on the AASLD practice guideline^49^. None of these HBV-infected patients had evidence of chronic comorbidities/conditions such as: autoimmune diseases, alcoholic liver disease, type 2 diabetes, cancers, addiction to smoking and alcohol. All patients were confirmed negative for anti-HCV and anti-HIV by ELISA assays. Laboratory parameters including HBV-DNA loads and liver function tests including alanine transaminase (ALT), aspartate transaminase (AST), total bilirubin and direct bilirubin, albumin, prothrombin were obtained from the patients’ medical records. In addition, 283 healthy individuals (HC) were collected and assigned as the control group. These individuals were healthy individuals who presented themselves to the hospital for a routine medical check-up. Hematological and biochemical parameters were normal and serological tests were negative for hepatitis C virus and human immune deficiency virus. Finally, plasma and blood cells were separated and frozen at − 80 °C until use.

### *PD-1* genotyping

Genomic DNA was isolated from 200 µl of whole blood using a DNA isolation kit (Qiagen, Hilden, Germany), following manufacturer’s instructions. The process of PD-1 genotyping was followed the study protocol as described^50^. The amplicon containing the variants *PD-1.5* (*rs2227981*) and *PD-1.9* (*rs2227982*) was amplified by PCR (PCR1) using the primer pairs PD-1.5/9_F: 5′-GCA AGA ATG CCA GGG ACA TTT CAG AG-3′ and PD-1.5/9_R: 5′-TGC CTG GTG CAG GTG CAG-3′. PCR amplification was performed out in 25 μl reaction volumes containing: 1 × PCR buffer, 0.2 mM dNTPs, 1 mM MgCl_2_, 0.15 mM of each primer, 1 unit of Taq polymerase and 50 ng of genomic DNA. Cycling conditions: denaturation at 95 °C for 5 min, followed by 35 cycles of three-step cycling with denaturation at 94 °C for 40 s, annealing at 66 °C (PCR1) and 60 °C (PCR2) for 40 s, and extension at 72 °C for 45 s and a final extension at 72 °C for 7 min. PCR products were purified using the Exo-SAP-IT PCR product cleanup reagent (Affymetrix Santa Clara, USA) 5 µl of purified PCR products were used as templates. Sequencing was performed using the BigDye terminator v.1.1 cycle sequencing kit (Applied Biosystems, Foster City, CA, USA) on an ABI 3130XL DNA sequencer according to the manufacturer’s instructions.

### Statistical analysis

All statistical analysis was performed using R version 3.1.2 (http://www.r-project.org). Statistical analysis plan was tailored as described previously^50^. In brief, genotype and allelic frequencies were determined by simple gene counting and the haplotype frequency was estimated using the expectation-maximum algorithm method implemented in the Arlequin v.3.5.2.2. The deviations from Hardy–Weinberg equilibrium were calculated for each group. We used a binary logistic regression adjusted for age and gender to analyze association of *PD-1* variants with HBV-related liver diseases applying for different genetic models and adjusted odds ratios (aOR) with 95% confidence intervals (CI) were calculated. Chi-square tests were used to test for significant differences of categorical variables and Mann–Whitney-Wilcoxon and Kruskal–Wallis tests were applied to compare quantitative variables between groups. Significance was set at a value of *p* < *0.05*.

## Results

### Baseline characteristics of HBV patients

**Figure 1:**
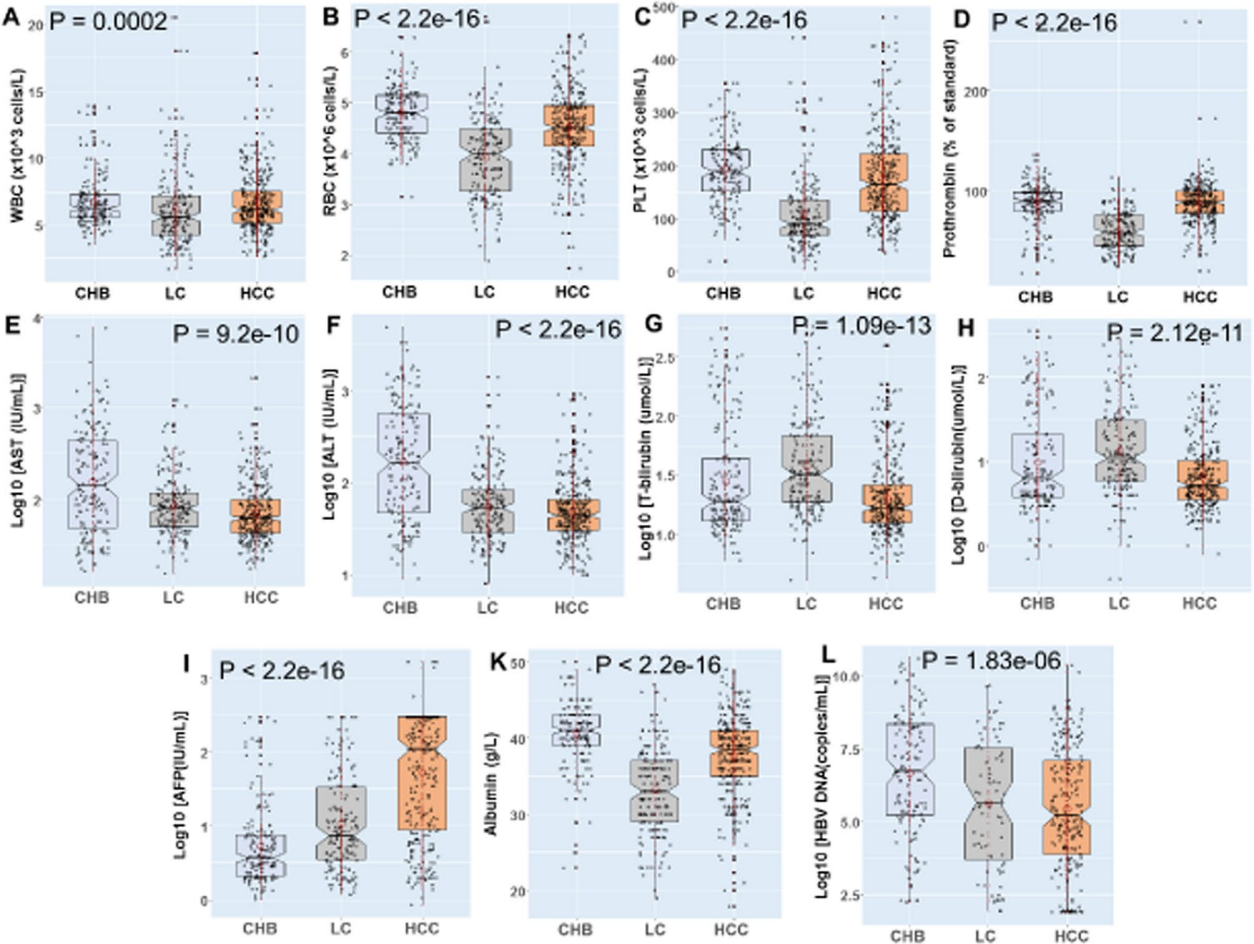
Evaluation of clinical parameters in HBV patient subgroups. *CHB* chronic hepatitis B, *LC* liver cirrhosis, *HCC* hepatocellular carcinoma, *PLT* platelets, *AST and ALT* aspartate and alanine amino transferase, *WBC* white blood cells, *RBC* red blood cells, *PLT* platelet, *IU* international unit. Box-plots illustrate medians with 25 and 75 percentiles with whiskers to 10 and 90 percentiles. *p-*values were calculated by Kruskal–Wallis test.

The baseline profiles of 682 HBV-infected patients and 283 healthy controls (HC) are described in Table 1 and Fig. 1. Most of the HBV and HC patients are male (88% and 63%, respectively) and the HCs were younger than HBV patients. The ALT, AST liver enzymes were significantly higher in CHB patients than other subgroups (*p* < 0.0001). Albumin and prothrombin levels, red blood cells and platelets were lower in LC patients than in other patient groups (*p* < 0.0001). HCC patients had higher AFP levels than CHB and LC patients (*p* < 0.001).

**Table 1:** Demographic and clinical characteristics of patients and controls.

Clinical characteristics	Patients (n = 682)	Controls (n = 283)	*p*
Age (years)	51 (15–90)	40 (15–69)	< 2.2e-16
Male/female	603/79	177/106	< 2.2e-16
HBsAg	Positive	Negative	NA
Anti-HCV	Negative	Negative	NA
Anti-HIV	Negative	Negative	NA
AFP (IU/mL)	10.8 (0.84–1660)	1.63 (0.82–8.86)	< 2.2e-16
HBV DNA (copies/mL)	6.8 × 10^5^ (10^2^–4.01 × 10^10^)	NA	NA
WBC (× 10^3^/L)	6.01 (1.69–20.5)	6.99 (3.85–13.2)	1.5e-6
RBC (× 10^6^/L)	4.5 (1.74–6.7)	4.79 (3.92–6.7)	4.4e-11
PLT (× 10^3^/L)	151 (3.7–479)	263 (118–422)	< 2.2e-16
AST (U/L)	73 (15–7700)	21 (12.2–59)	< 2.2e-16
ALT (U/L)	53.5 (8–4968)	18 (4–113)	< 2.2e-16
Total bilirubin (µmol/L)	19.9 (4.1–571)	11.2 (1.52–25.6)	< 0.0001
Direct bilirubin (µmol/L)	6.7 (0.4–349)	2.2 (1.8–21)	0.026
Albumin (g/L)	38 (18–86)	44.15 (17.8–47)	6.8e-14
Prothrombin (% of standard)	84 (17–269)	ND	NA

*AFP* aafa feto protei, *AST and ALT* aspartate and alanine amino transferase, *WBC* white blood cell, *RBC* red blood cell, *PLT* platelets. *IU* international unit, *NA* not applicable.

Values given are median and range. *P* values were calculated by Mann–Whitney–Wilcoxon and Chi-squared test where appropriate.

### *PD-1* variants and HBV related liver disease progression

The genotype and allele frequencies of two *PD-1* variants (*PD-1.5* and *PD-1.9*) in clinically classified 682 HBV patients and 283 HCs are summarized in the Tables 2 and 3. The variants were in Hardy–Weinberg equilibrium (*p* > 0.05) for both the cases and the controls. No significant association with HBV infection and clinical course was observed for *PD-1.5* variant. For *PD-1.9* variant, the heterozygous genotype *PD-1.9 CT* showed significant association with HBV infection (HBV patients *vs.* HC: OR = 1.4; 95% CI = 1.01–1.98; *p*_adj_ = 0.04).

**Table 2:** Association of *PD1* polymorphisms with susceptibility to HBV infection.

*PD1 *variants	HBV patients, n = 682 (%)	Healthy controls, n = 283 (%)	HBV patients vs. HC	
			OR (95% CI)	*p*
**PD-1.5 (rs2227981)—synonymous codon, upstream variant**				
*CC*	369 (54.1)	147 (51.9)	Reference	
*CT*	284 (41.6)	115 (40.6)	1.1 (0.8–1.5)	NS
*TT*	29 (4.3)	21 (7.4)	0.7 (0.4–1.5)	NS
**Allele**				
*C*	1022 (75)	409 (72.3)	Reference	
*T*	342 (25)	157 (27.7)	0.9 (0.7–1.1)	NS
**Dominant**				
*CC*	369 (54.1)	147 (51.9)	Reference	
*CT&TT*	313 (45.9)	136 (48.1)	1.0 (0.7–1.4)	NS
**Recessive**				
*CC&CT*	653 (95.7)	262 (92.6)	Reference	
*TT*	29 (4.3)	21 (7.4)	0.7 (0.4–1.5)	NS
**PD-1.9 (rs2227982)—missense, upstream variant**				
*CC*	219 (32.1)	110 (38.9)	Reference	
*CT*	345 (50.6)	125 (44.2)	**1.4 (1.01–1.98)**	**0.042**
*TT*	118 (17.3)	48 (17.0)	0.9 (0.6–1.54)	NS
**Allele**				
*C*	783 (57.4)	345 (61)	Reference	
*T*	581 (42.6)	221 (39)	1.1 (0.8–1.3)	NS
**Dominant**				
*CC*	219 (32.1)	110 (38.9)	Reference	
*CT&TT*	463 (67.9)	173 (61.1)	1.3 (0.9–1.8)	NS
**Recessive**				
*CC&CT*	564 (82.7)	235 (83)	Reference	
*TT*	118 (17.3)	48 (17)	0.8 (0.5–1.2)	NS

*OR* odds ratio, *n* number of chromosomes.

ORs and *P-*values were calculated by using binary logistic regression model adjusted for age and gender.

**Table 3:** Association of *PD1* polymorphisms with liver disease progression.

*PD1 *variants	CHB, n (%)	LC, n (%)	HCC, n (%)	LC vs. CHB		HCC vs. CHB		HCC vs. LC		HCC + LC vs. CHB		HCC vs. non-HCC	
				OR (95% CI)	*P*	OR (95% CI)	*P*	OR (95% CI	*P*	OR (95% CI)	*P*	OR (95% CI)	*P*
**PD-1.5 (rs2227981)—synonymous codon, upstream variant**													
*CC*	98 (50.8)	114 (62.3)	157 (51.3)	Reference		Reference		Reference		Reference		Reference	
*CT*	86 (44.6)	66 (36.1)	132 (43.1)	0.7 (0.4–1.1)	NS	1.01 (0.7–1.6)	NS	**1.5 (1.01–2.2)**	**0.009**	0.9 (0.6–1.3)	NS	1.3 (0.9–1.8)	NS
*TT*	9 (4.7)	3 (1.6)	17 (5.6)	0.3 (0.06–1.3)	NS	1.4 (0.6–3.8)	NS	**4.4 (1.2–15.6)**	**0.016**	0.9 (1.04–5.4)	NS	2.4 (0.4–2.34)	NS
**Allele**													
*C*	282 (73.)	294 (80.3)	446 (73)	Reference		Reference		Reference		Reference		Reference	
*T*	104 (27)	72 (19.7)	166 (27)	0.7 (0.5–1.02)	NS	1.1 (0.8–1.34)	NS	**1.6 (1.13–2.1)**	**0.006**	0.9 (0.7–1.3)	NS	1.3 (0.9–1.7)	NS
**Dominant**													
*CC*	98 (50.8)	114 (62.3)	157 (51.3)	Reference		Reference		Reference		Reference		Reference	
*CT&TT*	95 (49.2)	69 (37.7)	149 (48.7)	0.7 (0.4–1.1)	NS	1.05 (0.7–1.7)	NS	**1.6 (1.1–2.35)**	**0.013**	0.9 (0.6–1.3)	NS	1.3 (0.9–1.8)	NS
**Recessive**													
*CC&CT*	184 (95.3)	180 (98.4)	289 (94.4)	Reference		Reference		Reference		Reference		Reference	
*TT*	9 (4.7)	3 (1.6)	17 (5.6)	0.3 (0.07–1.5)	NS	1.4 (0.8–1.8)	NS	**3.7 (1.1–13.1)**	**0.02**	0.9 (0.4–2.4)	NS	2.1 (0.9–4.8)	NS
**PD-1.9 (rs2227982)—missense, upstream variant**													
*CC*	74 (38.3)	51 (27.9)	94 (30.7)	Reference		Reference		Reference		Reference		Reference	
*CT*	103 (53.4)	86 (47)	156 (51)	1.2 (0.7–2.0)	NS	1.3 (0.8–1.9)	NS	0.9 (0.6–1.5)	NS	1.2 (0.8–1.84)	NS	1.1 (0.8–1.6)	NS
*TT*	16 (8.3)	46 (25.1)	56 (18.3)	**3.8 (1.8–8.0)**	**0.0007**	**2.1 (1.01–4.3)**	**0.047**	0.6 (0.4–1.1)	NS	**2.9 (1.5–5.6)**	**0.004**	0.9 (0.6–1.6)	NS
**Allele**													
*C*	251 (65)	188 (51.4)	344 (56.2)	Reference		Reference		Reference		Reference		Reference	
*T*	135 (35)	178 (48.6)	268 (43.8)	**1.7 (1.2–2.4)**	**0.00014**	**1.3 (1.1—1.8)**	**0.029**	0.8 (0.6–1.1)	NS	**1.5 (1.1–2.0)**	**0.0043**	1.0 (0.8–1.2)	NS
**Dominant**													
*CC*	74 (38.3)	51 (27.9)	94 (30.7)	Reference		Reference		Reference		Reference		Reference	
*CT&TT*	119 (51.7)	132 (72.1)	212 (69.3)	1.6 (0.96–2.6)	NS	1.4 (0.9–2.1)	NS	0.8 (0.6–1.3)	NS	1.5 (0.98–2.2)	NS	1.1 (0.7–1.5)	NS
**Recessive**													
*CC&CT*	177 (91.7)	137 (74.9)	250 (81.7)	Reference		Reference		Reference		Reference		Reference	
*TT*	16 (8.3)	46 (25.1)	56 (18.3)	**3.4 (1.7–6.7)**	**0.00019**	**1.8 (1.09–3.6)**	**0.042**	0.6 (0.4–1.1)	NS	**2.5 (1.4–4.7)**	**0.0015**	0.9 (0.6–1.4)	NS

ORs and *P-*values were calculated by using binary logistic regression model adjusted for age and gender. Bold values present the statistical significance.

*CHB* chronic hepatitis B (n = 193), *LC* liver cirrhosis (n = 183), *HCC* hepatocellular carcinoma (n = 306), *non-HCC*  CHB + LC, *n*  number of chromosomes, *OR* odds ratio.

A significant difference in *PD-1.5* allelic and genotype frequencies was observed between HCC and LC patients [*PD-1.5* Allele T OR = 1.6 (1.13–2.1), *p*_adj_ = 0.006; *PD-1.5* genotype TT co-dominant model: OR = 4.4 (1.2–15.6), *p*_adj_ = 0.016; dominant model: OR = 1.6 (1.1–2.35), *p*_adj_ = 0.013; recessive model: OR = 3.7 (1.1–13.1), *p*_adj_ = 0.02]. For the *PD-1.9* variant significant differences in distribution of allele and genotypes were observed in LC, HCC and LC + HCC patients compared to CHB patients. The minor allele *PD-1.9 T* increased CHB patients’ risk towards disease progression either as LC, HCC or LC + HCC [LC vs. CHB: OR = 1.7(1.2–2.4), p_adj_ = 0.00014; HCC vs. CHB: OR = 1.3 (1.1–1.8), *p*_adj_ = 0.029; LC + HCC vs. CHB: OR = 1.5 (1.1–2.0); *p*_adj_ = 0.0043]. The *PD-1.9* homozygous genotype *TT* was observed frequently among patients with LC, HCC and LC + HCC groups compared to CHB patients in the co-dominant [LC vs. CHB: OR = 3.8(1.8–8.0), *p*_adj_ = 0.0007; HCC vs. CHB: OR = 2.1(1.01–4.3), *p*_adj_ = 0.047; LC + HCC vs. CHB: OR = 2.9(1.5–5.6), *p*_adj_ = 0.004] and recessive [LC vs. CHB: OR = 3.4(1.7–6.7), *p*_adj_ = 0.00019; HCC vs. CHB: OR = 1.8(1.09–3.6), *p*_adj_ = 0.042; LC + HCC vs. CHB: OR = 2.5(1.4–4.7), *p*_adj_ = 0.0015] models respectively.

### *PD-1* haplotypes and HBV-related liver diseases

A total of four haplotypes were reconstructed from both *PD-1.5* and *PD-1.9* variants and the frequencies were presented in the Table 4. No significant association in the distribution of haplotypes in HC and HBV and patient subgroups was observed. The analysis within HBV patient subgroups showed that the haplotype *CT* (*PD-1.5 C* and *PD-1.9 T*) was frequently observed in patients with advanced liver disease rather than those with CHB [LC vs. CHB: OR = 1.6(1.1–2.4), *p*_adj_ = 0.009; HCC vs. CHB: OR = 1.5 (1.1–2.2), *p*_adj_ = 0.016; LC + HCC vs. CHB: OR = 1.6(1.2–2.2), *p*_adj_ = 0.0028]. In addition, *haplotype TC* (*PD-1.5 T* and *PD-1.9 C*) was a prognostic indicator for HCC [HCC vs. non-HCC: OR = 1.4 (1.1–1.9); *p*_adj_ = 0.019]. Both *CT* and *TC* haplotypes contribute to liver disease progression and clinical outcome in HBV infected patients.

**Table 4:** Association of *PD1* haplotypes with HBV-related liver diseases.

Haplotypes (PD-1.5/PD-1.9)	HC (n, %)	CHB (n, %)	LC (n, %)	HCC (n, %)	LC vs. CHB		HCC vs. CHB		HCC + LC vs. CHB		HCC vs. Non-HCC	
	n = 566	n = 386	n = 366	n = 612	OR (95% CI)	*P*	OR (95% CI)	*P*	OR (95% CI)	*P*	OR (95% CI)	*P*
*CC*	192 (33.9)	148 (38.3)	117 (32)	179 (29.2)	Reference		Reference		Reference		Reference	
*CT*	217 (38.3)	134 (34.7)	177 (48.4)	267 (43.6)	**1.6 (1.1–2.4)**	**0.009**	**1.5 (1.1–2.2)**	**0.016**	**1.6 (1.2–2.2)**	**0.0028**	1.2 (0.9–1.5)	0.23
*TC*	153 (27)	103 (26.7)	71 (19.4)	165 (27.0)	0.9 (0.6–1.4)	0.6	1.4 (0.9–2.0)	0.09	1.2 (0.9–1.7)	0.3	**1.4 (1.1–1.9)**	**0.019**
*TT*	4 (0.7)	1 (0.3)	1 (0.2)	1 (0.2)	NA	NA	NA	NA	NA	NA	NA	NA

ORs and *P-*values were calculated by using binary logistic regression model adjusted for age and gender. Bold values present the statistical significance.

*CHB* chronic hepatitis B, *LC* liver cirrhosis, *HCC* hepatocellular carcinoma, *HC* healthy control, *non-HCC* CHB + LC, *n* number of chromosomes, *NA* not applicable, *OR* adjusted odds ratio.

### *PD-1* genetic variant and laboratory parameters

**Figure 2:**
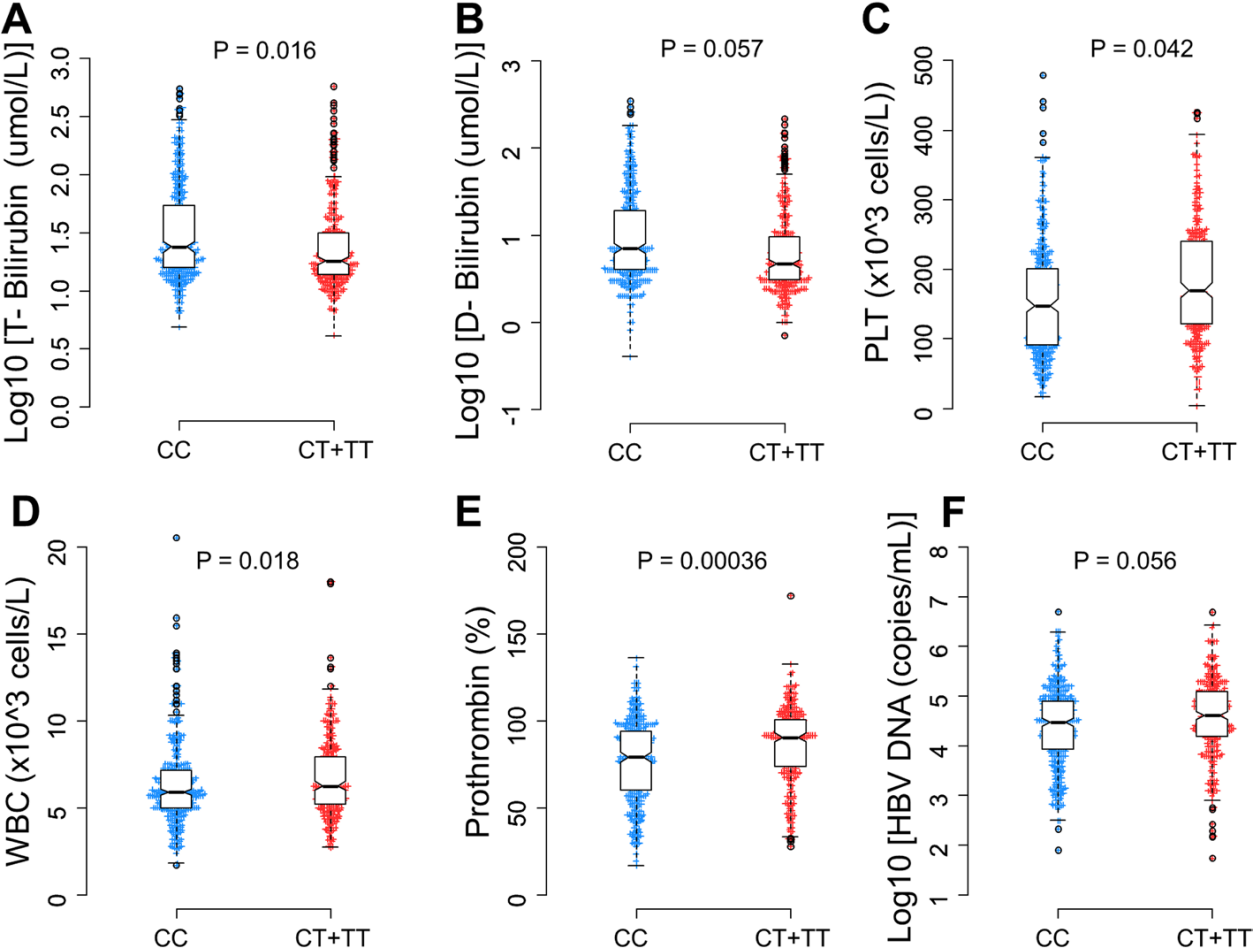
Association of *PD-1.5* variant with distinct laboratory parameters in HBV patients. Box-plots illustrate median values with 25 and 75 percentiles with whiskers to 10 and 90 percentiles; *p-*values were calculated by Mann–Whitney test.

**Figure 3:**
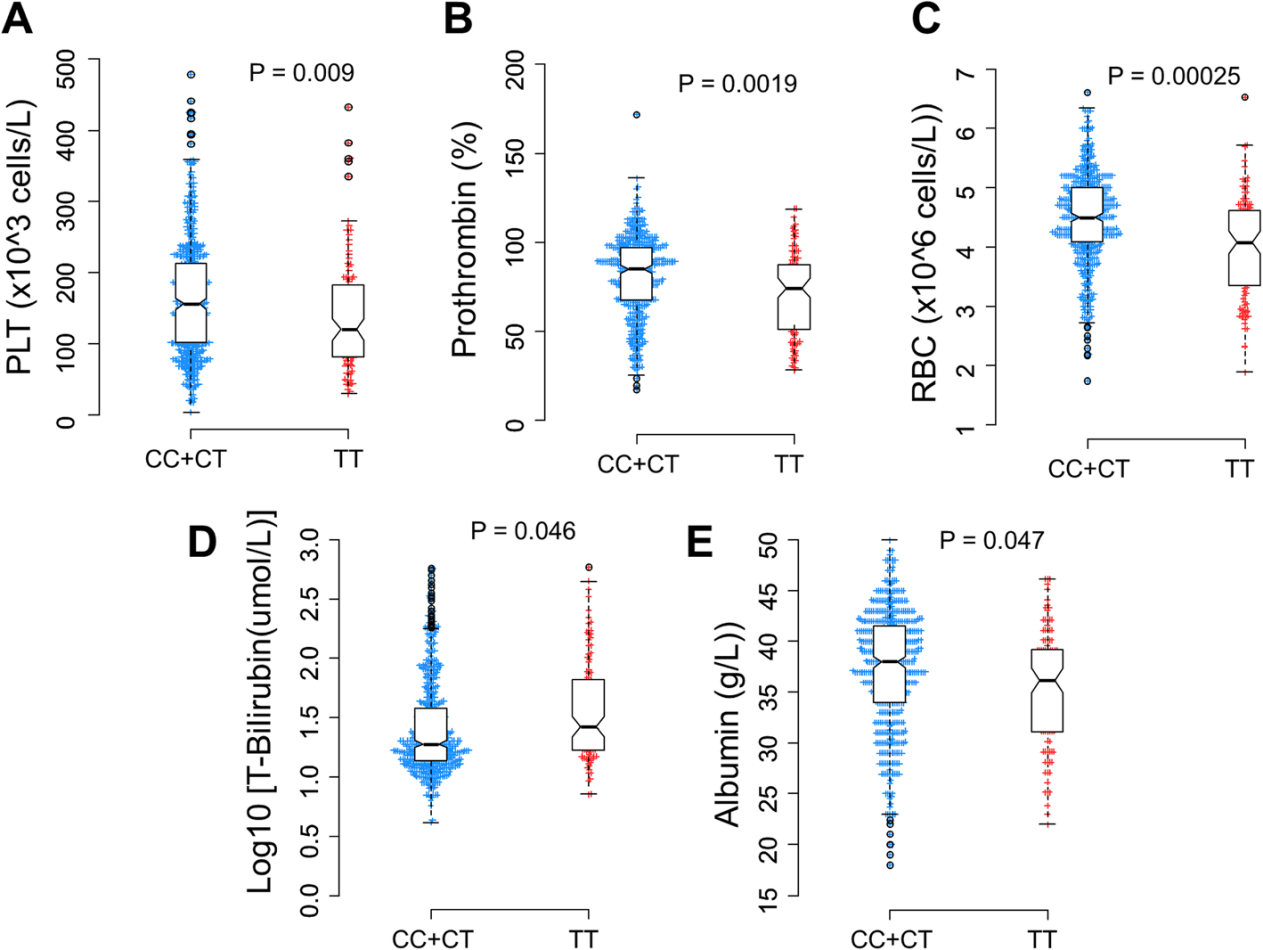
Association of *PD-1.9* variant with distinct laboratory parameters in HBV patients. Box-plots illustrate median values with 25 and 75 percentiles with whiskers to 10 and 90 percentiles; *p-*values were calculated by Mann–Whitney test.

The *PD-1.5* and *PD-1.9* genetic variants were associated with distinct biochemical and laboratory parameters (Figs. 2 and 3). The total and direct bilirubin levels were higher in patients with genotype *PD-1.5 CC* compared to those with genotypes CT/TT (*p* = 0.016 and *p* = 0.05, respectively). In addition, prothrombin levels were significantly decreased among those with *PD-1.5* CC genotypes *(p* = 0.00036). Equally other blood cell counts such as platelets, white and red blood cells were also significantly decreased among those carrying *PD-1.5* CC genotypes (*p* = 0.042, 0.018 0.056 respectively). In the *PD-1.9* variant, patients with the genotype *TT* had lower platelet, red blood cell, prothrombin and albumin levels compared to the CC/CT genotype (*p* = 0.009, 0.0019, 0.00025 and 0.047, respectively). In contrast, total bilirubin levels were significantly higher in the genotype *TT* than in the genotype *CC/CT*. There was no significant correlation between *PD-1.9* and other laboratory parameters such as liver enzymes, HBV DNA and AFP.

**Figure 4:**
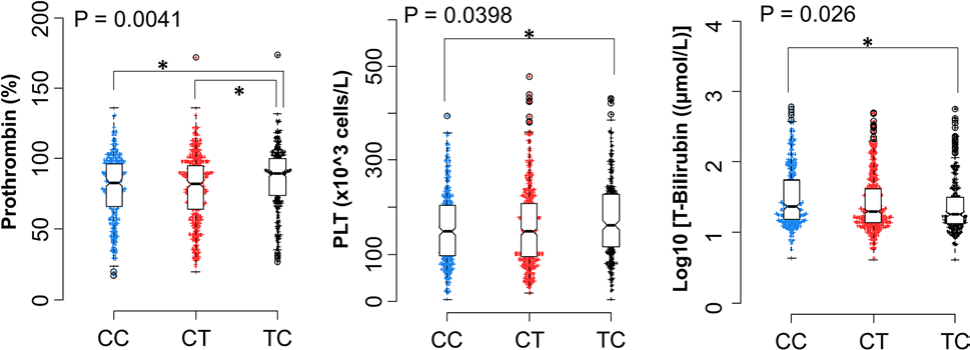
Association of *PD-1* haplotypes with distinct laboratory parameters in HBV patients. Box-plots illustrate median values with 25 and 75 percentiles with whiskers to 10 and 90 percentiles; *p-*values were calculated by Mann–Whitney test.

We also analyzed the association between constructed haplotypes and laboratory parameters (Fig. 4). We observed that patients with the TC haplotype had higher prothrombin and platelet levels than other *CC* and *CT* haplotypes (*p* = 0.0041, 0.0398, respectively). The *TC* haplotype was significantly associated with lower levels of total bilirubin compared to *CC* haplotypes (*p* = 0.026).

## Discussion

PD-1 is a member of the immunoglobulin superfamily and its cytoplasmic domain contain immunoreceptor tyrosine inhibitory motif associated with inhibitory signaling. The interaction between PD-1 and PD-L1 is known to contribute primarily to the depletion of T cells and thus involved in carcinogenesis. Several variants in the *PD-1* gene have been identified to associate with various infectious and autoimmune diseases, and cancers. In this case–control study, we investigated on the *PD-1.5 (*+ *7785T/C*) and *PD-1.9 (*+ *7625C/T)* variants in Vietnamese HBV-infected patients and controls. Two variants *PD-1.5* and *PD-1.9* were observed to influence the clinical outcome of HBV, in particular progression of liver disease.

*PD-1.5* is a synonymous substitution, whereas *PD-1.9* is a non-synonymous substitution (valine to alanine) that modulates structural and functional properties of PD-1. Several studies have investigated on these two variants with different human malignancies^31,32^, autoimmune disorders^51–53^ and infectious diseases, including HBV infection^41,47,54^. *PD-1.5* and *PD-1.9* variants susceptibility to HBV infection were earlier studied^47,54^, and in particular, the *PD-1.9 T* allele is considered as a predisposing factor for HBV susceptibility^47^. In addition, the frequency of *TT* and *CT* genotypes in HBV patients was significantly higher than healthy controls. Based on the reported results, Huang et al. pointed out that the CC genotype of rs2227982 and the C allele are protective factors in HBV infection^47^. In our study, we also reported that when analyzed with the age- and sex-adjusted logistic regression model, the frequency of *CT* genotype was significantly higher in HBV patients than in controls [co-dominant (*CT:CC*) model: OR = 1.4 (1.01–1.98), P = 0.042]. However, another study found no association between this variant and HBV infection^55^. For the *PD-1.5* polymorphism, we found no significant association with HBV infection. Our conclusions were in contrast to a previous study, which found an association between this polymorphism and susceptibility to chronic HBV infection in the Chinese population^54^. In addition, this polymorphism was also reported to be associated with HCV infection^56^.

Few studies suggest that *PD-1* as a convincing genetic indicator for different cancers in different world populations, however results were contradictory and ambiguous^31–35,46^. To date, comprehensive meta-analyses have been conducted to investigate the association of *PD-1* polymorphisms, notably *PD-1.5* and *PD-1.9*, with risk of cancer^32,46,57,58^. Concerning the *PD-1.5* polymorphism, Dong et al. showed that the *allele T* (compared to the *C* allele) is associated with a reduced risk of cancer in the general population^32^. In addition, two other meta-analyses have shown that the *PD-1.5 TT* genotype (*TT* compared to *CT/CC*) significantly reduces the overall risk of cancer {OR = 0.82, 95% CI = 0.68–0. 99, P = 0.04^57^]; [OR = 0.75, 95% CI: 0.64–0.86, P < 0.0001^46^} and {OR = 0.65, 95% CI: 0.47–0.90, P < 0.05,^58^}. In regards to *PD-1.9* polymorphism, no significant association between this variant and overall cancer susceptibility was established in these meta-analyses^32,46,57,58^. However, Hashemi et al.^57^ performed stratified analyses and indicated that the *PD-1.9* polymorphism was associated with increased risk of general cancer in hospital-based study (OR = 1.2, 95% CI = 1.05–1.37, P = 0.008, *CT/TT* vs. *CC*). Additionally, stratified analyses found the association between *PD-1.9* and increased risk of gastrointestinal cancer (OR = 1.16, 95% CI = 1.03–1.30, P = 0.017, *CT/TT* vs*. CC)* but decreased risk of breast cancer (OR = 0.73, 95% CI = 0.60–0.89, P = 0.002, *CT/TT* vs. *CC*).

It should be noted that these above meta-analyses included several case–control studies, but no studies have examined the association between *PD-1.5* and *PD-1.9* polymorphisms and HCC risk. To our knowledge, this is the first study in which we have examined the link between *PD-1.5* and *PD-1.9* polymorphisms and the progression of liver disease including HCC. We have shown that the *PD-1.5* and *PD-1.9* polymorphisms are associated with the progression of HBV-related liver diseases. Particularly, the variant *PD-1.9* shows a recessively homozygous advantage in disease progression and might be a risk factor for the advanced disease progression including LC and HCC. The results from our study indicate that *PD-1.9* polymorphism may influence liver disease progression and HCC development in chronic HBV infection. There is growing evidence that PD-1 overexpression is associated with T-cell dysfunction and exhaustion in chronic HBV infection and HCC development^59–61^.

As a single SNP site could not well represent the influence of *PD-1* genetic variants in HBV infection, we analyzed the association of haplotypes constructed from variants *PD-1.5* and *PD-1.9* with the liver disease progression and outcomes in HBV-infected patients. The study results could show a way by integrating different sites to completely elucidate the potent role of *PD-1* genetic variants in the risk of HBV infection and HBV-related clinical presentation. Still more works are needed to be done to examine the clinical significance of *PD-1* genetic variants as the host genetic perspective in HBV infection.

This study has two limitations. First, a longitudinal monitoring of HCC patients would be an added advantage in understanding the overall survival rates among patients with HBV-related HCC. Secondly, quantification of PD-1 gene expression in liver tissues of HCC patients may additionally warrant on the relevance these variants in clinical routine. Taken together, this study demonstrates on the role of *PD-1.5 and PD-1.9* genetic variants in predicting HBV infection risk and subsequent liver disease progression.
