# Author Correction: Genetic variants of programmed cell death 1 are associated with HBV infection and liver disease progression

**DOI:** 10.1038/s41598-022-06068-z

**Published:** 2022-02-01

**Authors:** Nghiem Xuan Hoan, Pham Thi Minh Huyen, Mai Thanh Binh, Ngo Tat Trung, Dao Phuong Giang, Bui Thuy Linh, Dang Thi Ngoc Dung, Srinivas Reddy Pallerla, Peter G. Kremsner, Thirumalaisamy P. Velavan, Mai Hong Bang, Le Huu Song

**Affiliations:** 1 Department of Blood-Borne Infectious Diseases, Institute of Clinical Infectious Diseases, 108 Institute of Clinical Medical and Pharmaceutical Sciences, Hanoi, Vietnam; 2 Vietnamese-German Center for Medical Research (VG-CARE), Hanoi, Vietnam; 3 Department of Biochemistry, 108 Institute of Clinical Medical and Pharmaceutical Sciences, Hanoi, Vietnam; 4 Faculty of Gastroenterology, 108 Institute of Clinical Medical and Pharmaceutical Sciences, Hanoi, Vietnam; 5 Centre for Genetic Consultation and Cancer Screening, 108 Institute of Clinical Medical and Pharmaceutical Sciences, Hanoi, Vietnam; 6 Hanoi Medical University, Hanoi, Vietnam; 7 Institute for Tropical Medicine, University Hospital Tübingen, Tübingen, Germany; 8 Centre de Recherches Médicales de Lambaréné (CERMEL), Lambaréné, Gabon

Correction to: *Scientific Reports* 10.1038/s41598-021-87537-9, published online 08 April 2021

In the original version of this Article, Peter G. Kremsner was incorrectly affiliated with ‘Vietnamese-German Center for Medical Research (VG-CARE), Hanoi, Vietnam’. Additionally, an affiliation was omitted. The correct affiliations are listed below.

Institute for Tropical Medicine, University Hospital Tübingen, Tübingen, Germany

Centre de Recherches Médicales de Lambaréné (CERMEL), Lambaréné, Gabon

The affiliations have been renumbered.

The original Article has been corrected.

